# Salvianolic Acid B Alleviates Limb Ischemia in Mice via Promoting SIRT1/PI3K/AKT Pathway-Mediated M2 Macrophage Polarization

**DOI:** 10.1155/2022/1112394

**Published:** 2022-05-24

**Authors:** Wenhao Niu, Feng Wu, Wenyue Cao, Yihong Chen, Yanda Zhang, Yasha Chen, Ru Ding, Chun Liang

**Affiliations:** ^1^Department of Cardiology, Second Affiliated Hospital of Naval Medical University, Shanghai 200003, China; ^2^Department of Cardiology, Yueyang Hospital of Integrated Traditional Chinese and Western Medicine, Shanghai University of Traditional Chinese Medicine, Shanghai 200437, China; ^3^Department of Ultrasonography, Shanghai Chest Hospital, Shanghai 200030, China; ^4^Department of Ultrasonography, Shanghai Chest Hospital, Shanghai Jiao Tong University, Shanghai 200030, China

## Abstract

Salvianolic acid B (Sal B) is an effective treatment agent for ischemic disease in China. However, Sal B's effects on peripheral arterial disease (PAD) and its mechanism remains poorly understood. Macrophage polarization plays a crucial role in PAD. Nevertheless, treatment modalities that increase the population of anti-inflammatory (M2) macrophages are limited. This study aimed to explore the protective effects of Sal B on limb perfusion and investigate the mechanism of Sal B-induced macrophage polarization. C57BL/6 male mice (6 weeks) were randomized into control, Model + NS, and Model + Sal B groups (*n* = 5). Then, we established a hind limb ischemia mouse model to assess the Sal B's role (15 mg/kg/d) in PAD. We quantified the blood perfusion via laser speckle contrast imaging (LSCI) and measured the capillary density and muscle edema with CD31 and H&E staining. The Sal B-induced macrophage polarization was confirmed by qPCR and ELISA. The results showed that the Sal B group exhibited a significant improvement in the blood perfusion, capillary density, muscle edema, and M2 markers gene expressions. Cell migration and tube formation were promoted in the endothelial cells stimulated with a culture supernatant from Sal B-treated macrophages. In contrast, endothelial functions improved by Sal B-treated macrophages were impaired in groups treated with SIRT1 and PI3K inhibitors. These findings provide evidence for Sal B's protective role in PAD and demonstrate the enhancement of macrophage polarization via the SIRT1/PI3K/AKT pathway.

## 1. Introduction

Peripheral arterial disease (PAD) is a major atherosclerotic complication which lowers the life expectancy and life quality of about 200 million people worldwide [1]. Insufficient arterial blood flow caused by atherosclerotic occlusions is one of the causes of clinical PAD symptoms and leads to pain at rest and nonhealing ulcers that may ultimately result in amputation [2]. Although most people with PAD do not have symptomatic claudication, they experience functional impairment that diminishes their quality of life [3, 4].

In experimental PAD, a growing body of evidence supports the theory that macrophages play a crucial role in vascular remodeling [5–8]. In the early stages of limb ischemia, pro-inflammatory and cytotoxic factors released by necrotic cells and tissues can recruit macrophages and transform them into classically activated macrophages (M1-polarized) to remove the cell fragments [9–12]. During the later phases of tissue repair, macrophages can transform into alternatively activated macrophages (M2-polarized) in response to IL-4 and IL-13 and contribute to tissue repair and regeneration [9, 10, 12, 13]. However, macrophages that infiltrate the ischemic muscle are likely to transform into M1-phenotype due to the high pro-inflammatory and cytotoxic milieu. Additionally, an increase in M1-macrophages and/or sustained M1-phenotype can significantly decrease neovascularization and perfusion recovery in ischemic tissues [13–15]. Thus, the regulation of macrophage polarization into M2 phenotype favors the shift toward neovascularization in distal ischemic muscle. However, therapies that could enhance the M2-macrophage population in ischemic muscle remain limited.

*Salvia miltiorrhiza* has been a common traditional Chinese medicine since ancient times [16]. Its roots have been used at neighborhood clinics to treat patients with thrombotic/ischemic diseases including coronary heart disease, acute cerebral ischemia-reperfusion, and thromboangiitis obliterans for decades through the inhibition of thrombosis and inflammatory responses [17, 18], Li et al., 2020 [19]. Our previous study found that Danhong injection (a Chinese material medica standardized product extracted from *Salvia miltiorrhiza* and *Flos Carthamus tinctorius*) could promote angiogenesis in diabetic mice after critical limb ischemia [20]. Salvianolic acid B (Sal B) is a bioactive component that is isolated from *Salvia miltiorrhiza*. Several studies have shown that Sal B mitigates ischemia symptoms in cardiovascular disease and I/R-induced cerebral injury by inhibiting oxidation and thrombosis [21–23]. It mitigates inflammatory factors as well. Sal B contributes to regulating inflammation and improves cardiac dysfunction in MI/R hearts by decreasing the quantity of M1-polarized macrophages via suppressing mTORC1-dependent glycolysis [24]. Sal B also inhibits inflammatory response in pulmonary fibrosis, which eventually provides symptom relief [25]. Moreover, Sal B exerts anti-inflammatory effects through regulating the Mincle-Syk-related pathway of macrophages analyzed by omics and transgenic methods [26]. In addition to modulating inflammation, many studies have revealed the relevance between Sal B and angiogenesis. For example, Sal B promotes angiogenesis and attenuates cardiac fibrosis and cardiac remodeling in diabetic cardiomyopathy [27]. Furthermore, angiogenesis and osteogenesis induced by Sal B-loaded chitosan/hydroxyapatite scaffolds can attenuate segmental bone defects [28]. Apart from this, Sal B exerts pro-angiogenesis by stimulating autophagy, which increases the random-pattern skin flaps' survival rate [29]. However, Sal B's effects and underlying mechanisms on limb ischemia and state of macrophage polarization in distal ischemic muscle remain unclear.

Thus, we investigated Sal B's effects on macrophage polarization in both *in vivo* limb ischemia models and *in vitro* mouse bone marrow-derived macrophages (BMDMs). Sal B exerts its anti-ischemia effects by promoting the M2 polarization of macrophages via the SIRT1/PI3K/AKT pathway. This indicates that Sal B is a therapeutic strategy for PAD.

## 2. Materials and Methods

### 2.1. Chemicals and Reagents

Sal B (purity: 99.73%, Cat. No. S4735), EX527 (SIRT1 inhibitor), and LY294002 (PI3K inhibitor) were purchased from Select (Houston, TX, USA). CD31 immunohistochemical staining antibody, western blot antibodies (SIRT1, AKT, phospho-AKT (Ser473 and ThrT308)), and FACS antibodies (CD11b and F4/80) were bought from Cell Signaling Technology (Danvers, MA, USA). Antibodies to PI3 Kinase p85 and phospho-PI3 Kinase p85 were purchased from Zen-Bioscience (Chengdu, China). RIPA lysis buffer, protease, and phosphatase inhibitor cocktail were purchased from Beyotime Biotechnology (Shanghai, China); a BCA Protein Assay Kit was bought from Thermo Fisher Scientific (Carlsbad, CA, USA); a secondary antibody was bought from ZSGB-BIO (Beijing, China); and the ECL reagent was purchased from Millipore (MA, USA). RNAiso and PrimeScript RT Master Mix were brought from TaKaRa (TaKaRa, Tokyo, Japan); an SYBR Green PCR Kit was purchased from Roche (Switzerland). DMEM, FPS, and penicillin-streptomycin were bought from Thermo Fisher (Carlsbad, CA, USA); recombinant murine M-CSF was purchased from R&D Systems (Emeryville, CA, USA). IL4 was bought from Sigma-Aldrich Chemie GmbH (Steinheim, Germany). ECM medium and ECGS were brought from Sciencell (CA, USA). The Matrigel matrix was obtained from Corning (MA, USA). Interleukin (IL)-6 and IL-10 ELISA assay kits were purchased from Multisciences (Hangzhou, China).

### 2.2. Animals

We purchased male C57BL/6J mice aged 5 weeks from Ji Hui Experimental Animal Breeding Company (Shanghai, China). Mice were acclimated for at least 1 week and kept in a temperature- and humidity-controlled room with a 12-h light/dark cycle. The mice were allowed *ad libitum* access to a standard diet and water.

All of the experimental procedures were approved by the Ethics Review of Animal Use Application at Naval Medical University (license number SYXK (HU) 2017-004). The study was conducted in accordance with the National Institutes of Health guide for the care and use of laboratory animals. All operations were performed under anesthesia, and we undertook all possible efforts to minimize suffering in accordance with the ARRIVE guidelines on animal research.

### 2.3. Establishing a Hind Limb Ischemia Model and Drug Treatment

We randomized one group of mice into a control group, and the other mice underwent hind limb ischemia surgery following adaptive feeding for 1 week. Model mice were anesthetized using intraperitoneal sodium pentobarbital (60 mg/kg body weight). A limb ischemia model was established as described in a previous report [30]. Model mice were then randomly allocated into either a Model + NS group or a Model + Sal B group that was intraperitoneally injected either normal saline or Sal B for 12 d before the mice were sacrificed. The Sal B's optimal intraperitoneal injection concentration was 15 mg/kg/d in accordance with the results of preliminary experiments. Sham-operated animals in control group underwent the same procedures without limb ischemia.

### 2.4. Hind Limb Ischemia Monitored with Laser Speckle Contrast Imaging (LSCI)

We anesthetized the mice and placed them on a heating blanket at 37°C postoperatively. The LSCI system (Perimed Instruments AB, Sweden) was used before, immediately after, and on days 4, 8, and 12 after the operation. The blood flow recovery ratio = ischemic limb perfusion (left hind limb)/nonischemic limb perfusion (right hind limb) × 100%.

### 2.5. Histopathology Analysis and Immunohistochemical Staining

Twelve days after the ischemic surgery, we sacrificed the mice with an overdose of isoflurane, and harvested the hind limbs. Then, we stored them in liquid nitrogen for further research. For histopathologic analysis, we fixed the tissues with formalin, embedded them with paraffin, and cut them into 3-*μ*m-thick sections. Muscle cell injury scores were evaluated using H&E staining, as previous reported [31]. We randomly selected five fields of vision at 50 × magnification in tissue sections of every mouse. The proportion of injured muscle cells = injured muscle cells/total number of muscle cells × 100% and we calculated the average proportion of injured muscle cells.

Vascularity of gastrocnemius muscles from mice was assessed by immunohistochemical staining using CD31 referring to a previous research [32]. After anesthesia, the animals were sacrificed and the gastrocnemius muscles were harvested and fixed in 4% paraformaldehyde. Then, we cut them into 3-*μ*m-thick sections for CD31 immunohistochemical staining. We randomly selected five fields of vision at 100 × magnification in tissue sections of every mouse, and CD31 staining was quantified using the ImageJ software. We recorded the ratio the intervention to control group.

### 2.6. Preparing the BMDMs, HUVECs, and Cell Cultures

The mouse BMDMs (bone marrow-derived macrophages) were isolated from the femur and the tibia bone marrow cells, and cultured in DMEM supplemented with 10% FPS, 1% penicillin-streptomycin and 20 ng/mL recombinant murine M-CSF at 37°C and 5% CO_2_ for 7 days. To determine cell purity, we conducted FACS analysis of CD11b and F4/80. Thereafter, the macrophages were divided into a control group, Sal B groups (at concentrations of 1, 5, and 25 *μ*M for 24 h) and an IL4 group (10 ng/mL for 24 h).

The HUVECs were purchased from American Type Culture Collection (ATCC, VA, USA) and cultured in a ECM medium with P/S and ECGS. The cells were cultured in a humidified incubator at 37°C in an atmosphere with 5% CO_2_. In order to explore whether the Sal B-treated macrophage cell culture supernatant could stimulate cell migration and tube formation of HUVECs, we divided the HUVEC groups into a control group, Sal B (25 *μ*M for 24 h) group, macrophage cell culture supernatant group (the BMDMs were cultured for 24 h and then collected culture supernatant to culture HUVECs for 24 h), and Sal B-treated macrophage cell culture supernatant group (the BMDMs were treated with Sal B at 25 *μ*M for 24 h and then collected culture supernatant to culture HUVECs for 24 h). Moreover, in order to verify whether the SIRT1/PI3K/AKT pathway was indispensable to the cell migration and tube formation of HUVECs induced by the Sal B-treated macrophage cell culture supernatant, we divided the HUVEC groups into a control group, Sal B group, Sal B-treated macrophage cell culture supernatant group, Sal B+EX527-treated macrophage cell culture supernatant group (the BMDMs were pretreated with EX527 at 10 *μ*M for 6 h, and then treated with Sal B 25 *μ*M for 24 h. Next, we collected the culture supernatant to culture HUVECs for 24 h), and the Sal B+LY294002-treated macrophage cell culture supernatant group (the BMDMs were pretreated with LY294002 at 10 *μ*M for 6 h, and then treated with Sal B 25 *μ*M for 24 h. Next, we collected the culture supernatant to culture HUVECs for 24 h).

### 2.7. Western Blot Analysis

On day 12, we collected the mice's left limb gastrocnemius tissues in each group. The tissue homogenate was achieved with a tissue homogenizer (NewZongKe, China) after adding RIPA lysis buffer, and quantified using the BCA Protein Assay Kit. The supernatant was collected following 2 cycles of centrifugation. Then, we used equal amounts of protein (20–30 *μ*g) for SDS-PAGE electrophoresis and then transferred it to a PVDF membrane. Next, we blocked the PVDF membrane with 5% skim milk for 1 h at room temperature and added SIRT1, PI3K, phospho-PI3K, AKT, phospho-AKT (Ser473 and ThrT308), and GAPDH antibodies at 4°C overnight. After performing PBST cleaning three times for 10 min each, the corresponding secondary antibody was added to incubate for 1 h. Finally, we did three cycles of PBST washing for 10 min each. The proteins in the PVDF membranes were enhanced with the ECL reagent and detected with UVP BioImaging systems (CA, USA).

### 2.8. Quantitative Real-Time PCR Assay

We added RNAiso to the gastrocnemius tissue in order to homogenize for RNA extraction. Then, we reverse-transcribed RNA to cDNA to explore gene expressions using the PrimeScript RT Master Mix. This was followed by qRT-PCR using a Roche SYBR Green PCR Kit. The relative gene expression levels were calculated with equation 2−^ΔΔCt^. The primers used in this study are described in Table 1.

### 2.9. Enzyme-Linked Immunosorbent Assay (ELISA)

Serum specimens from the experimental animals and culture media from BMDMs were analyzed to quantify the level of IL-6 and IL-10 using ELISA assay kits following the manufacturer's instructions. The absorbance at 450 nm was measured using a microplate reader (BioTek, USA).

### 2.10. Tube Formation Assay

100 *μ*l of the Matrigel matrix (Corning, MA, USA) was loaded in each well of a 96-well plate and then incubated at 37°C for 30 min. Then, we seeded HUVECs on the pre-coated wells at a density of 5–7 × 10^4^ cells/well and cultured for 24 h at 37°C in a tissue culture incubator. Images of tube morphology were taken using an inverted microscope (Leica, DFC290HD, Germany) at × 100 magnification.

### 2.11. Cell Migration Assay

Migration was evaluated by a transwell assay. Cells were resuspended in 200 *μ*l of the serum-free ECM medium and seeded on the upper chambers of transwell inserts with 8 *μ*m pore size (Corning, MA, USA). The ECM-conditioned medium supplemented with 10% FBS was added to the lower chamber of the transwells. After incubation for 24 h, any cells remaining on the upper transwell membrane were removed with a cotton tip. Then, we fixed the migrated cells with 4% paraformaldehyde for 20 min and stained them with 0.5% crystal violet for 20 min. The average number of cells was counted in five random fields.

### 2.12. Microarray Data Mining and Kyoto Encyclopedia of Genes and Genomes (KEGG) Pathway Analysis

We analyzed differentially expressed genes (DEGs) for the various SIRT1 knockout tissues microarray data using the GEO2R Online Analytical tool. The DEGs were further analyzed using the KEGG pathway enrichment analysis. Genes related to SIRT1 were obtained from the STRING database (https://string-db.org/). We conducted the visualization of SIRT1 pathways and the related genes with the Metascape database (http://metascape.org/).

### 2.13. Molecular Docking of Sal B with SIRT1

We used ChemOffice to construct 3D structures of the Sal B. Then, we minimized the chemical constituents' energies with MMFF94 force field. RCSB Protein Data Bank (PDB) was used to obtain the SalB 3D structure in PDB format. We used PyMOL to analyze protein dehydration, hydrogenation, and other operations. Next, we used AutoDock to convert the compounds and target protein format to PDBQT format. Finally, we ran AutoDock Vina for virtual docking.

## 3. Statistical Analysis

All of the data are expressed as mean ± standard deviation (SD) values. We performed multiple comparisons with one-way analysis of variance (ANOVA) using SPSS 22.0 (SPSS Inc., USA). The *p* value < 0.05 was considered statistically significant.

## 4. Results

### 4.1. Sal B Protected against Limb Ischemia

**Figure 1:**
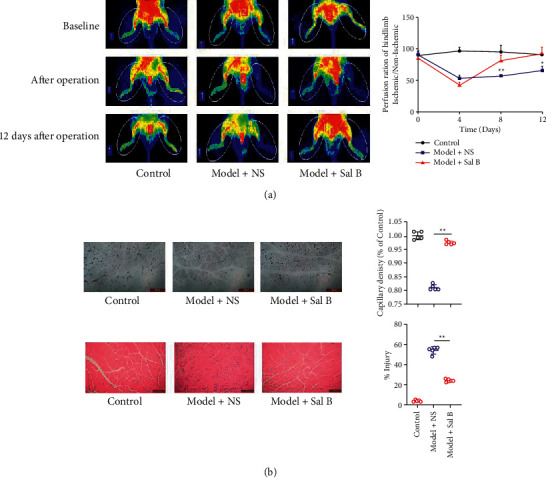
Sal B protected against limb ischemia. (a) Representative photographs of perfusion recovery in ischemic hind limbs. (b) Representative photographs of CD31 immunohistochemistry and HE staining in the ischemic hind limbs (each group *n* = 5; in Figure (a), ^*∗*^ represents the Model + NS group compared with the Model + Sal B group; ^*∗*^*P* < 0.05, ^*∗∗*^*P* < 0.01).

We investigated the link between Sal B and limb ischemia improvement in model mice. The blood flow showed impairment on the ischemic side after the operation, as assessed with LSCI. Compared with the Model + NS group, the Sal B-treated model mice displayed better blood perfusion in the ischemic limb (Figure 1(a)). In summary, this result showed that Sal B improved ischemic limb perfusion, which was consistent with our previous work [20].

### 4.2. Sal B Improved Angiogenesis Repair and Attenuated Muscle Edema in Ischemic Limbs in Mice

Next, we investigated Sal B's effects on angiogenesis repair and muscle edema. We harvested gastrocnemius muscle from the left hind limb in each group. The capillary density was significantly higher in the Model + Sal B group than in the Model + NS group (Figure 1(b)). The mice from the Model + NS group showed muscle fiber edema and cell injury, compared to the rounded and intact borders of the muscle fibers in the control group and Model + Sal B group. Moreover, the proportion of injured muscle cells in the Model + Sal B group was significantly lower than that in the Model + NS group (Figure 1(b)). These results suggest that Sal B increased angiogenesis repair and reduced muscle edema.

### 4.3. Sal B Promoted M2 Polarization In Vivo and In Vitro

**Figure 2:**
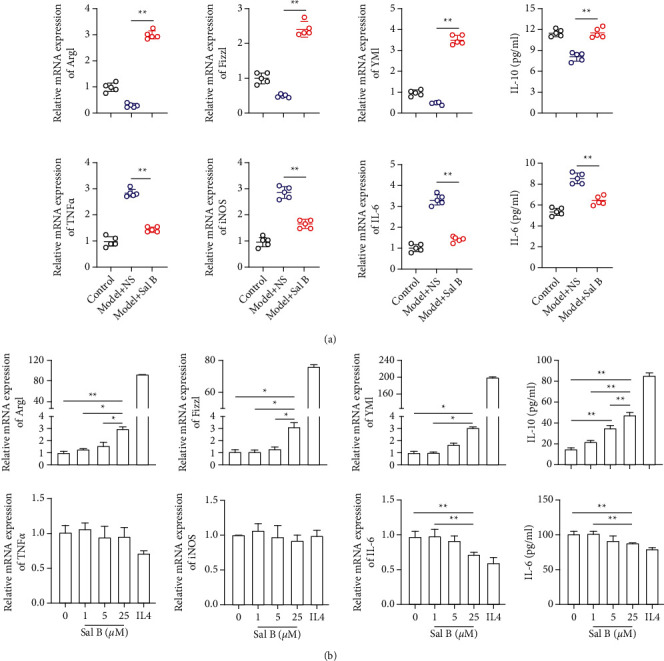
Sal B promoted M2 polarization *in vivo* and *in vitro*. (a) mRNA and protein level of M1 and M2 makers expression showed that Sal B contributed to M2 polarization and inhibited M1 polarization in vivo. (b) In vitro qPCR and ELISA assays showed that the expression of M2 markers were higher in the Sal B (25 *μ*M) group than that in control group (in the animal experiment, each group *n* = 5; ^*∗*^*P* < 0.05, ^*∗∗*^*P* < 0.01).

Macrophage polarization is crucial to the pathological progression of limb ischemia. In order to confirm macrophage polarization in limb ischemia model mice treated with Sal B, we used qPCR assay to analyze the expression of M1 macrophage pro-inflammatory markers and M2 anti-inflammatory markers, both *in vivo* and *in vitro*. The expression of M1 macrophage markers in the Model + NS group was significantly higher than that in the Model + Sal B group. In contrast, the anti-inflammatory macrophages' infiltration was significantly higher in the Model + Sal B group than in the Model + NS group. The isolated plasma showed that the level of IL-6 was significantly higher in Model + NS group compared with the Model + Sal B group. Moreover, The level of IL-10 was significantly higher in the Model + Sal B group (Figure 2(a)). Consistently, the expression of M2 markers in the Sal B (25 *μ*M) group was significantly higher than that in control group in cell experiments (Figure 2(b)).

### 4.4. Sal B-Treated Macrophage Cell Culture Supernatant Increased Cell Migration and Tube Formation in HUVECs

**Figure 3:**
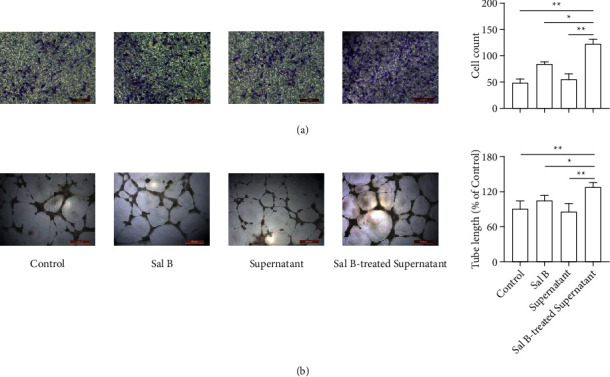
Cell migration and tube formation of HUVECs increased in the Sal B-treated macrophage cell culture supernatant group. (a) Sal B-treated macrophage cell culture supernatant could increase cell migration in endothelial cells. (b) Ability of tube formation in endothelial cells could be promoted in cell culture supernatant group (^*∗*^*P* < 0.05, ^*∗∗*^*P* < 0.01).

To explore the effect of Sal B-induced M2-polarized macrophage on cell migration and tube formation in HUVECs, we used Sal B-treated macrophage cell culture supernatant to treat HUVECs. The results showed greater cell migration in the Sal B-treated macrophage cell culture supernatant group (Figure 3(a)). Then, we tested whether Sal B-treated macrophage cell culture supernatant promoted HUVEC tube formation *in vitro*. The results showed that cell culture supernatant increased the tube formation in endothelial cells (Figure 3(b)). In sum, these results showed that Sal B-induced M2-polarized macrophage cell culture supernatant promoted cell migration and tube formation in HUVECs.

### 4.5. Sal B Increased M2 Polarization via SIRT1 Upregulation, Both In Vivo and In Vitro

**Figure 4:**
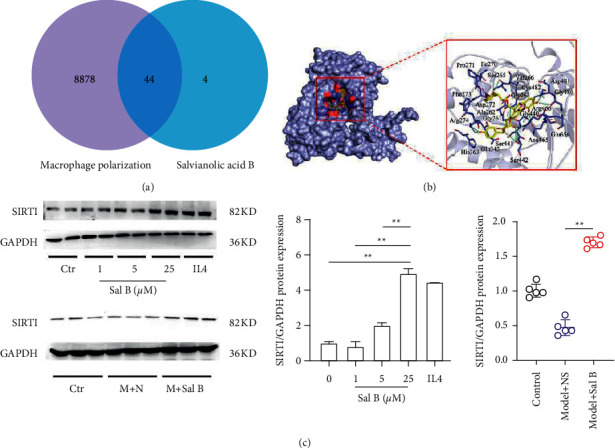
Elevation of the SIRT1 in Sal B-induced M2 polarization *in vivo* and *in vitro*. (a) Venn diagram showed that 44 genes had relationship with both macrophage polarization and Sal B. (b) Schematic diagrams showed the Sal B-binding sites. (c) SIRT1 protein levels in mice and BMDMs (in the animal experiment, each group *n* = 5; ^*∗*^*P* < 0.05, ^*∗∗*^*P* < 0.01).

We used the GeneCards database (http://www.genecards.org/) to identify genes related to macrophage polarization and Sal B, and screened out 44 genes through the Venn diagram (Figure 4(a)). Next, we used molecular docking to evaluate the binding affinity between Sal B and proteins translated from those genes above. Among them, we chose SIRT1 for further verification due to its great binding affinity with Sal B (−12.8 kcal/mol, Table 2). The binding structure between Sal B and SIRT1 is shown in Figure 4(b). Finally, we verified that Sal B upregulated SIRT1, both *in vivo* and *in vitro* (Figure 4(c)).

### 4.6. Sal B Upregulated PI3K/AKT Pathway, In Vivo and In Vitro

In order to explore the mechanisms through which SIRT1 induced M2 polarization, we analyzed DEGs in various SIRT1 knockout tissues' microarray data with the GEO2R online analytical tool, and used the KEGG pathway enrichment analysis to analyze the DEGs. We found that the PI3K/AKT pathway was downregulated in SIRT1 knockout tissues, indicating that the PI3K/AKT pathway was a potential target pathway that required further research [33, 34] (Supplementary Figure 1A). Next, STRING analysis showed SIRT1's close interaction with other genes. Molecules related to the PI3K/AKT pathway were colored in red, molecules related to the regulation of immune system processes were colored in green, molecules related to the activation of innate immune response were colored in yellow, and molecules related to the positive regulation of immune response were colored in purple (Supplementary Figure 1B) [3]. Furthermore, analysis of the metascape database showed that genes obtained from the STRING database had close relationships with the PI3K/AKT pathway as well (Supplementary Figures 1C, 1D, 1E).

**Figure 5:**
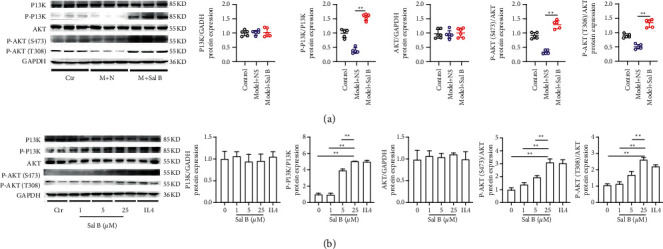
Sal B upregulated the PI3K/AKT pathway both *in vivo* and *in vitro*. (a) Protein level of the PI3K/AKT pathway in mice. (b) Protein level of the PI3K/AKT pathway in BMDMs (in the animal experiment, each group *n* = 5; ^*∗*^*P* < 0.05, ^*∗∗*^*P* < 0.01).

Thus, we examined PI3K/AKT pathway's involvement in Sal B-induced M2 polarization. We analyzed the key effectors, phosphor-PI3K, and phosphor-AKT (Ser473 and Thr308) in the PI3K/AKT pathway with western blot assay. The expression of phosphor-PI3K and phosphor-AKT in the Model + Sal B group was significantly higher than that in the Model + NS group (Figure 5(a)). In line with mouse findings, *in vitro* cell experiments showed that the expression of the PI3K/AKT pathway in the Sal B (25 *μ*M) group exceeded that of the control group (Figure 5(b)). These results indicated that Sal B increased M2 polarization via the upregulation of the SIRT1/PI3K/AKT pathway, both *in vivo* and *in vitro*.

### 4.7. SIRT1/PI3K/AKT Pathway Was Indispensable to the Cell Migration and Tube Formation of HUVECs Induced by Sal B-Treated Macrophage Cell Culture Supernatant

**Figure 6:**
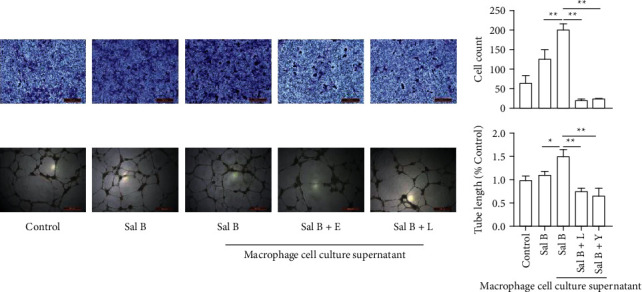
Cell migration and tube formation in HUVECs decreased in the Sal B+EX527 or Sal B+LY294002-treated macrophage cell culture supernatant groups ^*∗*^*P* < 0.05, ^*∗∗*^*P* < 0.01.

The results showed that cell migration in HUVECs decreased in the Sal B+EX527 and LY294002-treated macrophage cell culture supernatant groups (Figure 6(a)). Then, we used tube formation assay to show that Sal B+EX527 or LY294002-treated macrophage cell culture supernatant could attenuate tube formation in HUVECs *in vitro* (Figure 6(b)).

## 5. Discussion

The recovery of blood perfusion in the skeletal muscle of PAD depends on the interaction between multiple cell types [14, 35, 36]. The macrophages' polarization state affects this process, especially in diseased tissues [8, 37]. However, the macrophages that infiltrate the ischemic muscle tend to be of a pro-inflammatory type, owing to the highly inflammatory and ischemic environment [13, 14]. Hence, therapies which increase M2-like-macrophages' population or preserve the M2-like-phenotypeare are more likely to be successful PAD treatments.

As an active water-soluble component of *Salvia miltiorrhiza*, Sal B benefits patients with ischemic disease via antiapoptosis, antioxidant, and inflammatory regulation [38, 39]. Previous studies have shown that Sal B can also increase the anti-inflammatory macrophage population [24, 38]. In the current study, we showed that Sal B caused a significant improvement in blood perfusion of the ischemic limb. This leads to angiogenesis and protects muscle cells.

Additionally, the mice and BMDM experiments showed that Sal B significantly increased the expression of M2 macrophage markers. Therefore, Sal B appears to have the potential to be a novel therapeutic for PAD.

SIRT1, a nicotine adenine dinucleotide dependent deacetylase, regulates several pathophysiological processes and plays a crucial role in the cell cycle, aging, and metabolism [40,41]. Previous studies have demonstrated that SIRT1 could regulate tissue repair [42, 43]. Furthermore, inhibition of SIRT1 decreases M2 phenotype polarization via the downregulation of autophagy and the aggravation of oxidative stress [44, 45]. Several reports have also investigated Sal B's effect on anti-inflammatory effects via SIRT1 [46, 47]. Previous reports have also shown that SIRT1 can regulate the PI3K/AKT/STAT6 pathway leading to M2 macrophage polarization (L et al. 2019). Moreover, it has been demonstrated that SIRT1/PI3K/AKT pathway regulates proliferation, apoptosis, and various other pathophysiological processes as well [48–50]. In this study, to further investigate the underlying mechanism, we analyzed the expression of the SIRT1 and PI3K/AKT pathway. We found that Sal B increased the expressions of the SIRT1 and the PI3K/AKT pathway, both *in vivo* and *in vitro*. Thus, Sal B contributes to M2 phenotype polarization via the SIRT1/PI3K/AKT pathway.

**Figure 7:**
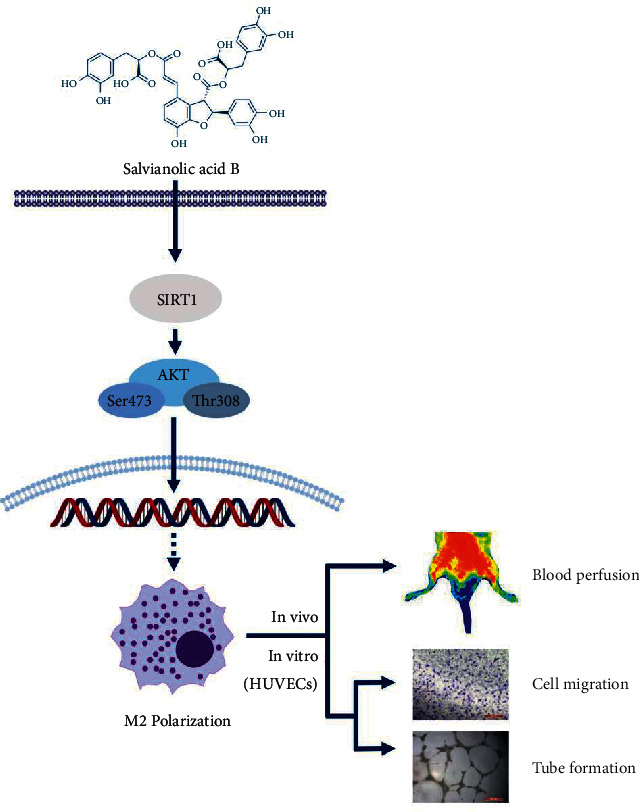
Schematic drawing summarizing Sal B's pharmacological mechanisms in limb ischemia treatment.

In order to verify our findings, we tested whether the SIRT1/PI3K/AKT pathway was required for cell migration and tube formation of HUVECs. Our results showed that cell migration and tube formation decreased in the Sal B+EX527 and LY294002-treated macrophage cell culture supernatant groups. Previous studies found that EX527 increased NF-*κ*B p65 acetylation through the inhibition of SIRT, leading to the macrophage inflammatory responses [51]. Moreover, EX527 administration abolished the M2 phenotype polarization through inhibition of autophagy [45]. LY294002 could decrease the phosphorylation of both AKT and STAT3 followed by inhibition of M2 phenotype polarization [52]. Furthermore, suppression of PI3K/AKT signaling with LY294002 can cause a dramatic downregulation of M2 polarization in disease outcome [53]. These results highlight the importance of the SIRT1/PI3K/AKT pathway in reducing inflammatory stress and angiogenesis (Figure 7).

## 6. Conclusion

Our results showed that Sal B, a promising drug for PAD, improved limb ischemia via M2 macrophage polarization by the SIRT1/PI3K/AKT pathway, thereby exerting an antilimb ischemia effect.

## Figures and Tables

**Table 1:** Sequence of primers used for quantitative real-time PCR.

Gene	Forward sequence (5'⟶3′)	Reverse sequence (5'⟶3′)
TNF*α*	GAGTGACAAGCCTGTAGCC	CTCCTGGTATGAGATAGCAAA
iNOS	CACCAAGCTGAACTTGAGCG	CGTGGCTTTGGGCTCCTC
IL6	GCAATGGCAATTCTGATTGTATG	AAGGACTCTGGCTTTGTCTTTCT
Arg1	CCAGAAGAATGGAAGAGTCAGTGT	GCAGATATGCAGGGAGTCACC
Fizz1	CTGCCCTGCTGGGATGACT	CATCATATCAAAGCTGGGTTCTCC
YM1	CAAGTTGAAGGCTCAGTGGCTC	CAAATCATTGTGTAAAGCTCCTCTC
GAPDH	TCACCACCATGGAGAAGGC	GCTAAGCAGTTGGTGGTGCA

**Table 2:** Binding affinity between Sal B and proteins.

Protein name	Binding affinity (kcal/mol)	Protein name	Binding affinity (kcal/mol)
MYC	−16	EDN1	−7
SIRT1	−13	SHC1	−6.5
ABCB1	−12	MMP1	−6.4
MTOR	−11.4	NR1I2	−6.2
CASP3	−11	AKR1B1	−6.1
MAPK1	−10.7	EIF4EBP1	−6
CTNNB1	−10.5	HSPA5	−5.9
PPARA	−10.3	ATF4	−5.6
NOS3	−10	PHB	−5.4
VCAM1	−9.7	SMAD3	−5.3
MMP2	−9.3	ATF2	−5.1
ACE	−9.1	AIFM1	−5
MAPK8	−9	BMP7	−4.7
MAPK14	−8.8	EIF2AK3	−4.3
TGFB1	−8.6	PPIA	−4
MAPK3	−8.4	SIK2	−3.8
MET	−8.3	VLDLR	−3.5
HMGCR	−8	PRMT1	−3.3
CREB1	−7.5	TAGLN2	−3.3
ACHE	−7.4	CDH5	−2
RB1	−7.2	ERP29	−1
SERPINE1	−7.1		

## Data Availability

All data included in this study are available upon request by contacting the corresponding author.

## Supplementary Materials

Supplementary Materials Supplementary figure 1: PI3K/AKT pathway was regulated by SIRT1.
