# Supplementary figures and images for: Salvianolic Acid B Alleviates Limb Ischemia in Mice via Promoting SIRT1/PI3K/AKT Pathway-Mediated M2 Macrophage Polarization
Source: Evid Based Complement Alternat Med. 2022 May 24;2022:1112394. doi: 10.1155/2022/1112394 (PMC9155924; doi:10.1155/2022/1112394)

A

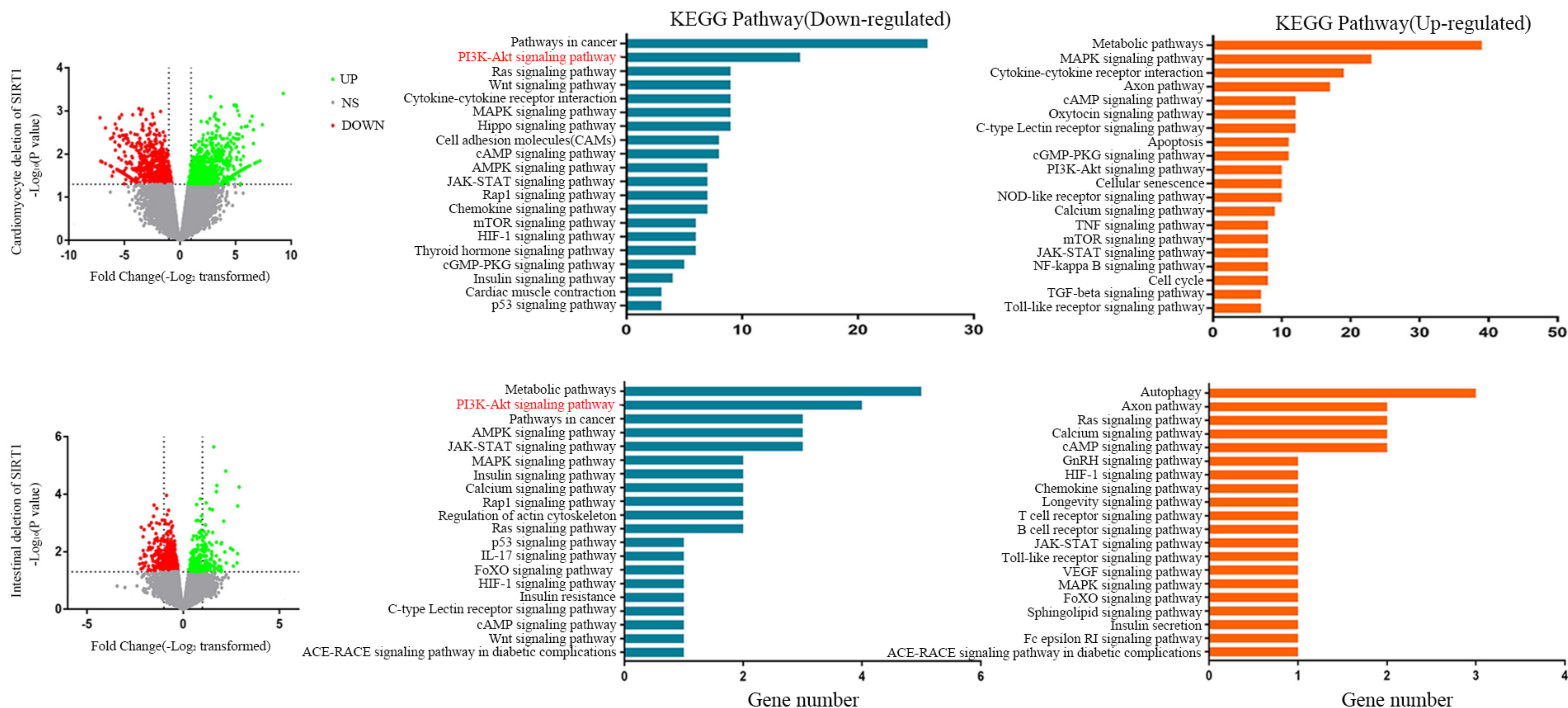

B

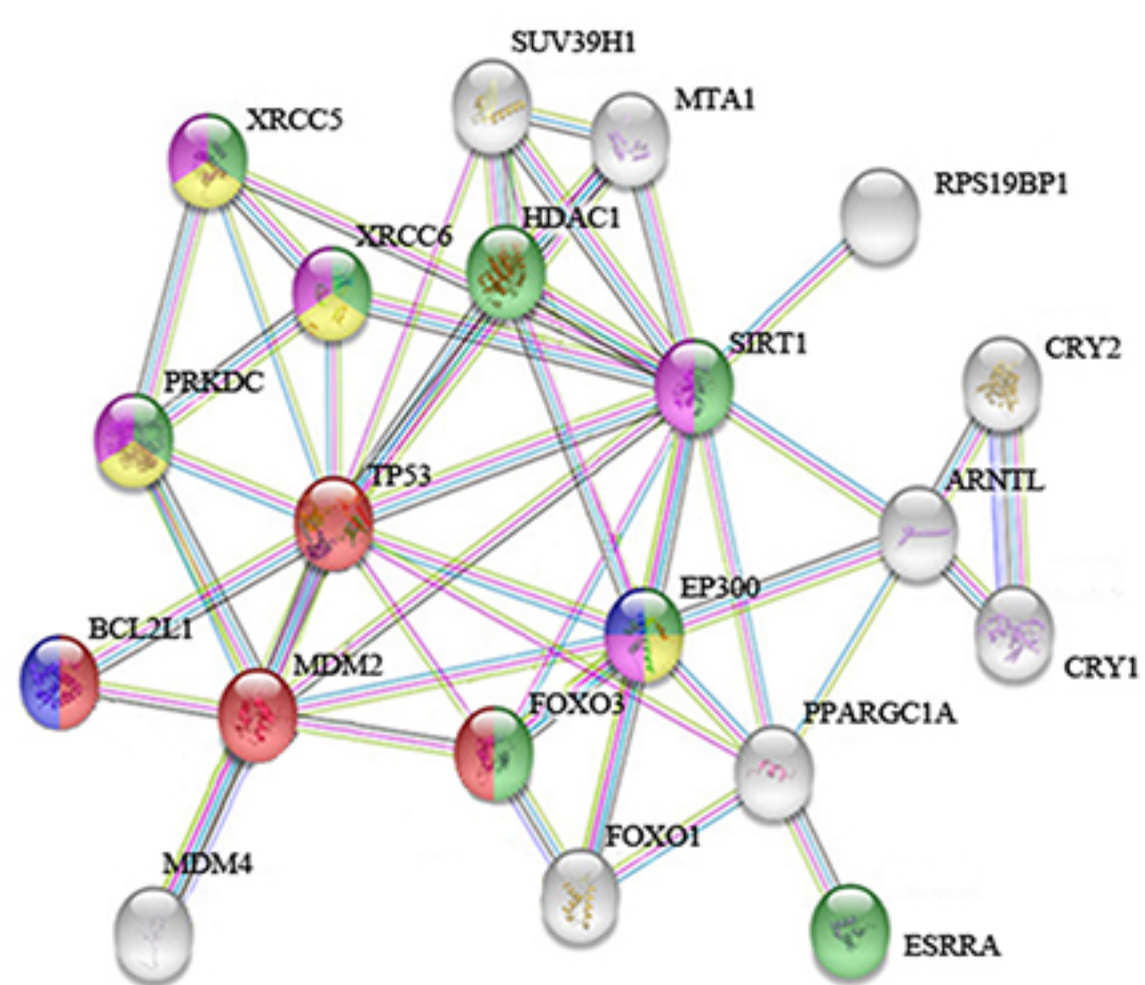

C

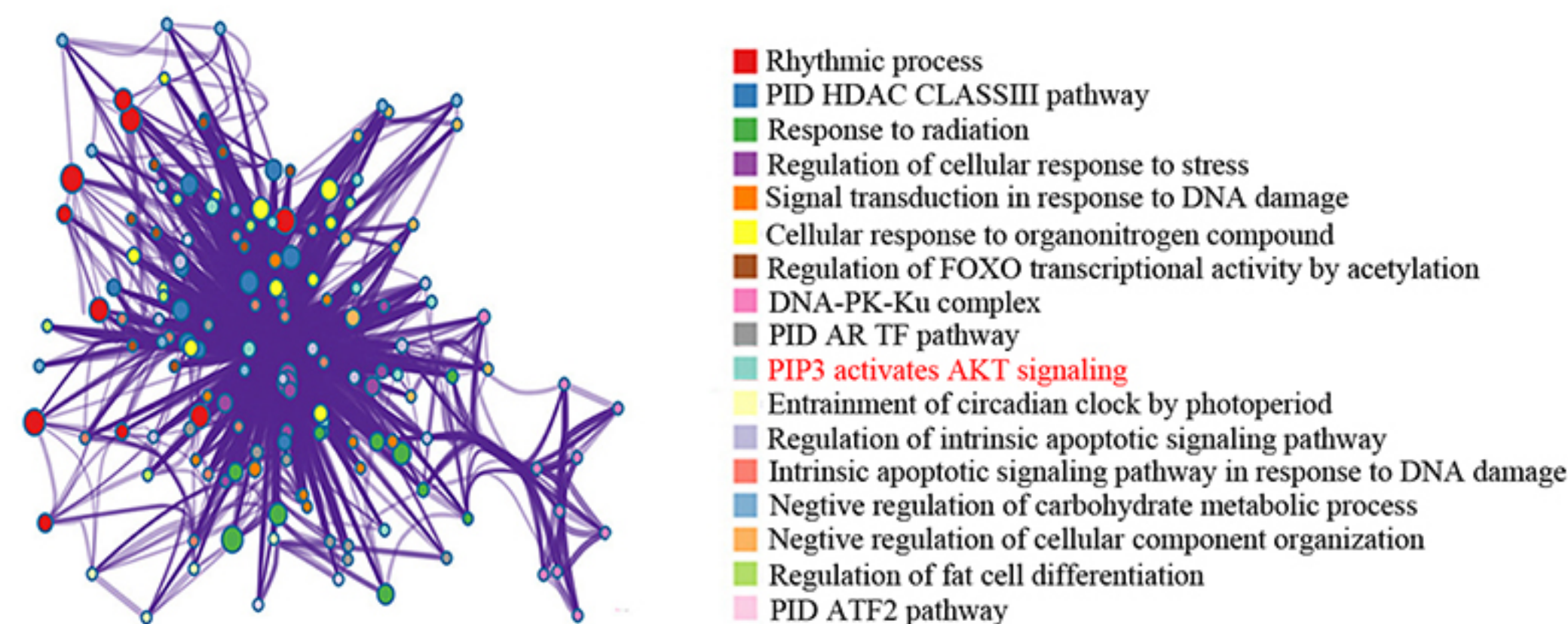

D

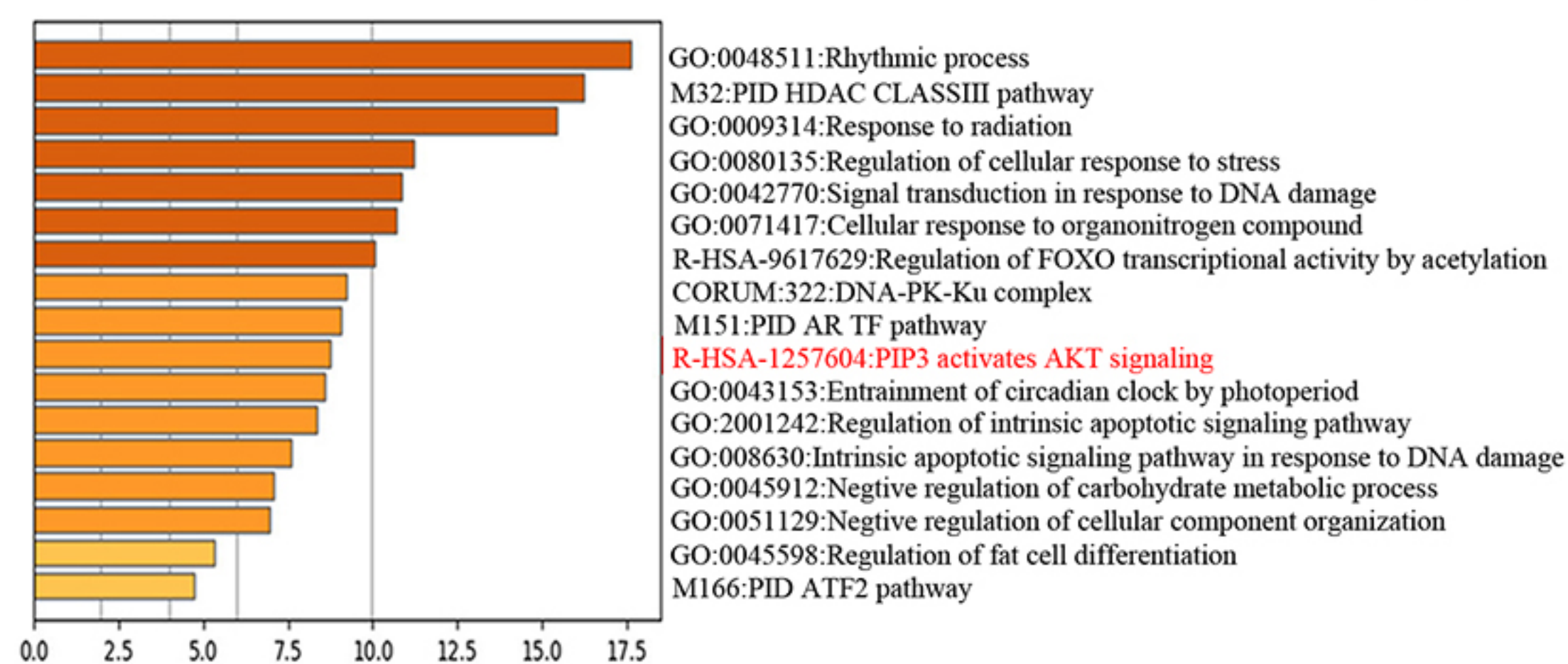

E

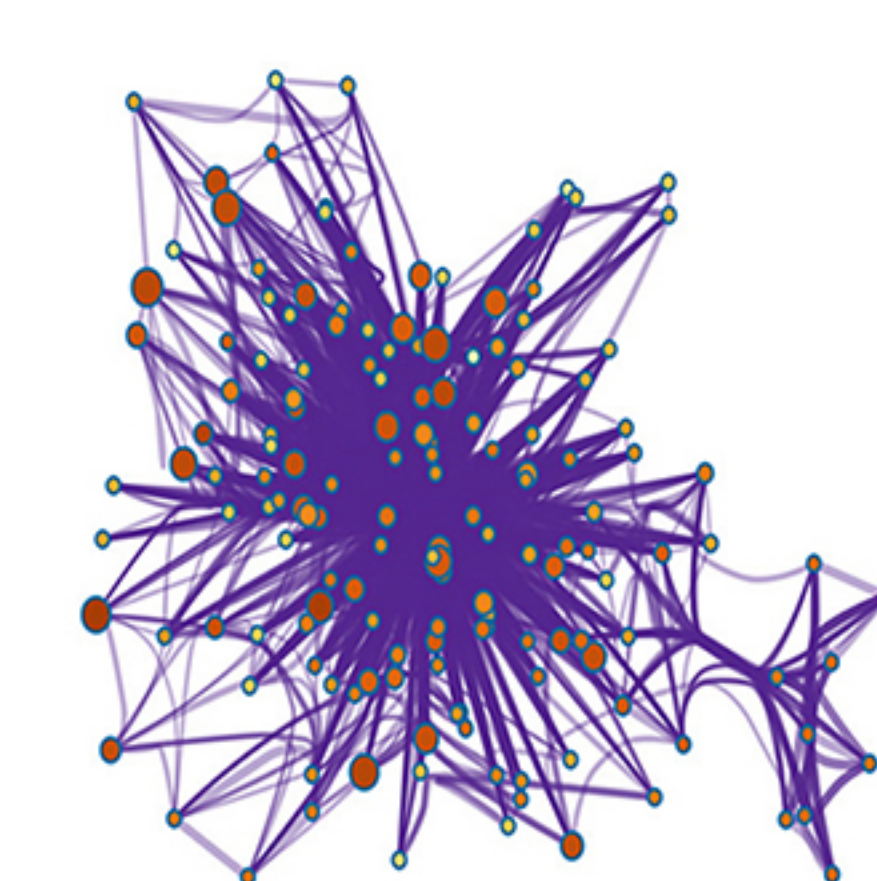

Supplement: Supplementary Materials — Supplementary figure 1: PI3K/AKT pathway was regulated by SIRT1. [file 1112394.f1.pdf]
